# Uniform patterns based area-efficient and accurate stochastic computing finite impulse response filter

**DOI:** 10.1371/journal.pone.0245943

**Published:** 2021-01-27

**Authors:** Muhammad Ijaz, Syed Azhar Ali Zaidi, Aamir Rashid

**Affiliations:** Electronics Engineering Department, University of Engineering and Technology, Taxila, Pakistan; Taipei Medical University, TAIWAN

## Abstract

Stochastic computing has recently gained attention due to its low hardware complexity and better fault tolerance against soft errors. However, stochastic computing based circuits suffer from different errors which affect the output accuracy of these circuits. In this paper, an accurate and area-efficient stochastic computing based digital finite impulse response filter is designed. In the proposed work, constant uniform patterns are used as stochastic numbers for the select lines of different MUXes in the filter and the error performance of filter is analysed. Based on the error performance, the combinations of these patterns are proposed for reducing the output error of stochastic computing based filters. The architectures for generating these uniform patterns are also proposed. Results show that the proposed design methodology has better error performance and comparable hardware complexity as compared to the state-of-the-art implementations.

## 1 Introduction

Stochastic computing (SC) is an unconventional computing technique, where, the numbers are represented as probabilities in a bit-stream. It was first introduced in 1960s [1] as a low cost alternative to binary computing, since it requires simple logic circuits for performing complex arithmetic operations [2]. SC also has the advantage of inherent fault tolerance against the transient and soft errors that is highly desirable in today’s deep sub-micron technology. Due to these advantages, SC is used in many applications such as neural networks, low-density parity check (LDPC) codes, machine learning, image processing, control systems, etc. [3–12].

Recently, SC is used for the implementation of digital filters [13], since, a digital filter is a key component in many of the above mentioned applications. A digital filter, especially, a higher order filter, requires large number of adders and multipliers and therefore, occupies a significant area in the hardware implementation of these applications. SC is employed to implement a low-cost and low-power implementation of digital filters. In SC, an addition operation between two stochastic numbers is implemented with the help of a multiplexer and the multiplication operation is implemented with a single logic gate (AND/XOR). Therefore, SC-based filters can be implemented with the help of simple logic gates and a tree of multiplexers. This results in very simple circuits for digital filters. However, SC-based digital filters suffer from correlation errors which reduces the accuracy of these filters. In SC, a stochastic number generator (SNG) is used, containing a random number generator (RNG), such as a linear-feedback shift register (LFSR), to convert a deterministic number into a stochastic number (SN). In order to generate highly uncorrelated SNs for all the inputs and coefficients of SC-based filters, a large number of RNGs are required. Many prior works have implemented SC-based digital finite impulse response (FIR) filters using large number of RNGs and therefore, these RNGs consume as much as 90% of their circuit area. E.g. in [14] SC-based FIR filter is designed using three different approaches and the area, power consumption, delay and the accuracy is compared with the conventional binary circuits. The hard-wired weighted average (HWA) approach in their work consumes less area then the other two approaches, however, the SNGs and counters in all their approaches use as much as 85% of the total area. In [15] a non-scaled stochastic adder is designed to increase the accuracy of the conventional SC-based FIR filters. However, the proposed circuit consumes 2.45 times more area as compared to the conventional SC-based approach for a 63rd order FIR filter.

In order to reduce the area of SC-based filters, a RNG is shared among many SNGs. However, this produces highly correlated bit streams which reduces the accuracy of these circuits. The authors in [16] have employed a design technique based on the sharing of RNGs for reducing the area of SC-based FIR filters. Two circuits are proposed in [16], namely, *same-depth-share* circuit and *all-share* circuit. The *same-depth-share* circuit shares one LFSR with all the SNGs of MUXes that have same-depth in the circuit (where, depth of a MUX is defined as the number of MUXes in the path from the output of Filter to that MUX). The *same-depth-share* case greatly reduces the area of SC-based filters, however, the number of RNGs in *same-depth-share* circuit increases with the increase in the order of the filter. The *all-share* circuit shares one RNG with all the SNGs used in the circuit, where, the output of RNG is circularly shifted with different shift amounts used at inputs and each consecutive stage of the MUX tree. The *all-share* case requires only 1 LFSR irrespective of the filter order. However, the accuracy of *all-share* case depends on the shift amounts applied at different stages and in most of the cases has reduced accuracy as compared to the *same-depth-share* case. More recent work [17] has suggested a permutation based technique in which the authors find the permutations of the RNG output with minimum average correlation for sharing with different SNGs used in the circuit. The achieved mean square error in their work is better than [16] for *all-share* case, however, the accuracy is still reduced as compared to *same-depth-share* case.

In this paper, a design strategy based on the constant uniform patterns is proposed. Different constant uniform patterns are applied at the select lines of MUXes and their impact on the error performance of FIR filters is analyzed. Based on the error performance, the combinations of these constant patterns are proposed for reducing the error of filters. The architectures for generating these uniform patterns are also proposed. Results show that the proposed design methodology achieves higher accuracy and reduced area as compared to the *same-depth-share* case for all implemented FIR filters of different orders. The hardware complexity is also comparable to the *all-share* case for all order FIR filters.

The rest of the paper is organized as follows. Section 2 introduces the basic theory of SC and the types of errors encountered in SC. Section 3 discusses the basic theory of SC-based FIR filters. In section 4, the proposed design methodology is discussed, and the results of the error analysis are given. The proposed architectures for constant patterns generation are also given in section 4. Section 5 presents the experimental results of the proposed design methodology. Section 6 concludes the paper.

## 2 Basics of stochastic computing

A stochastic number *A* is a sequence of binary bits of length *K* with *K*1 one’s and *K* − *K*1 zeros. The value represented by the SN is the probability of any bit to be 1 in the bit stream and this value corresponds to the deterministic number *c*. The two well-known formats of SNs are *unipolar* and *bipolar* format [2]. In a unipolar format, the probability of one’s in stochastic number is equal to *c*, i.e. *P*(*A*) = *K*1/*K* = *c*, where *c* ∈ [0, 1]. In a bipolar format, the probability of one’s in SN is equal to (*c* + 1)/2, and therefore, *c* lies in the range of -1 to +1. E.g. a number 0.333 can be represented in 6-bits as 101000 (unipolar format) or 110011 (bipolar format). The representation of a number in stochastic domain is not unique, and in general, there are $\left(\begin{array}{c} K1\\ K \end{array}\right)$ different possible combinations of SN corresponding to a real number *c*.

**Fig 1:**
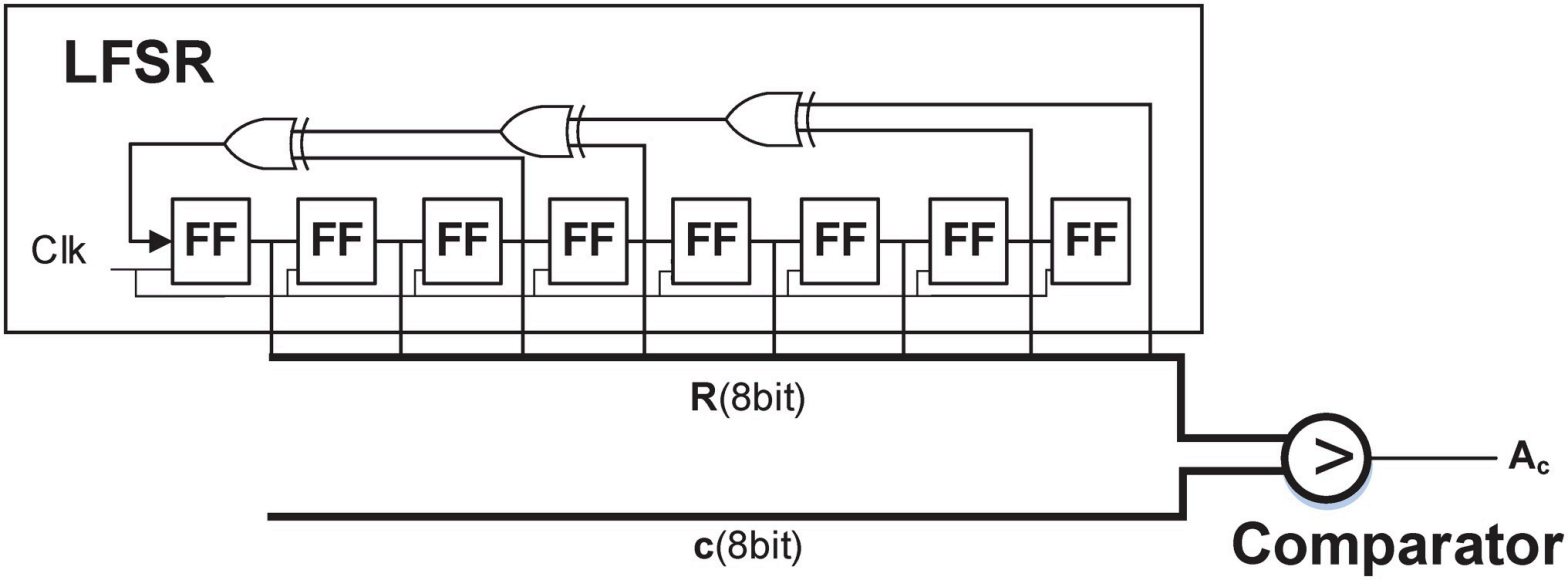
Stochastic number generator (SNG).

**Fig 2:**
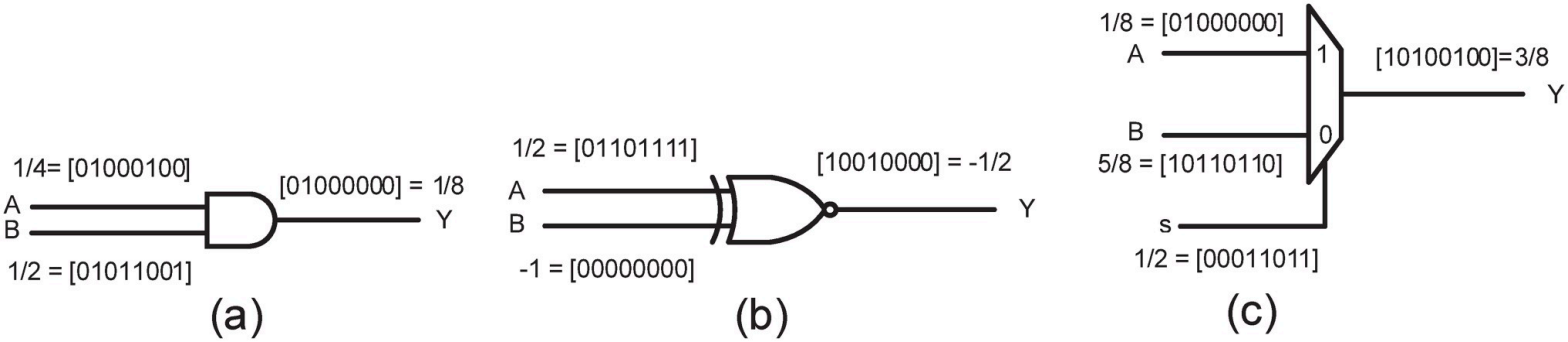
SC basic components: (a) Unipolar multiplier, (b) Bipolar multiplier, (c) Weighted adder.

The conversion from deterministic to SN is done by using a SNG. The SNG contains a pseudo random number generator, such as LFSR, for generating a *k*−bit random number in each clock cycle and a comparator for comparing the *k*−bit random number with the *k*-bit deterministic number *c*. The comparator produces a 1 if the random number is less than *c* and a 0 otherwise. Fig 1 shows an LFSR based SNG. The SN is converted back into the deterministic number by using a counter. Different arithmetic operations on SNs can be done by using simple logic circuits. Some of the basic building blocks of stochastic logic circuits are shown in Fig 2. E.g. multiplication of two SNs can be done by using a single AND gate in unipolar format, i.e. P(*A*1)×P(*A*2) ≅ P(*A*_1_∧ ≅ *A*_2_) = P(*a*1_1_ ∧ *a*2_1_ *a*1_2_ ∧ *a*2_2_ *a*1_3_ ∧ *a*2_3_…*a*1_*i*_ ∧ *a*2_*i*_), where, *a*1_*i*_ and *a*2_*i*_ are the *i*^*th*^ bits of the stochastic numbers *A*1 and *A*2 and ∧ denotes the logical AND operation between the bits. Similarly, a MUX can be used to perform weighted addition in stochastic computing. Consider two SNs *A* and *B* applied at the inputs 0 and 1 of MUX, respectively and a SN *S* applied at the select line of MUX. The MUX performs the following operation: (1 − P(*S*)).P(*A*) + P(*S*).P(*B*) ≅ (1 − *s*) × *c*1 + *s* × *c*2, where, *s*, *c*1 and *c*2 are the deterministic numbers and *S*, *A* and *B* are their corresponding SNs. The scaled addition is performed in order to keep the sum less than or equal to 1. E.g. for *s* = 0.5 the the output of MUX will be *c*1 + *c*2/2. However, this scaling results in inaccurate results, especially for the addition of large number of SNs. A better scaled addition circuit is discussed in section 3. An example of weighted addition based on unipolar format is shown in Fig 2(c). Since the digital filters are based on weighted additions, therefore, the MUX plays an important role in SC-based digital filters, where the weighted additions are implemented with the help of a MUX tree.

### 2.1 Errors in stochastic computing

The accuracy in stochastic computing is affected by several errors, such as, rounding error, conversion error and correlation error. The rounding errors occur due to the minimum precision of SNs which depends on the length *K* of SN. Increasing the length of SNs results in the reduction of rounding errors and vice versa. In this paper, the length of SNs is fixed to *K* = 2^*k*^, where, *k* is the bit-width of deterministic number, and therefore, the effect of rounding errors on the accuracy of SC is not analyzed.

As mentioned above, the conversion of deterministic number to stochastic is done with the help of SNG, which usually contains a *k*−bit pseudo-random number generator, such as, an LFSR. The number of 1’s produced in the SN by SNG is equal to the deterministic number. The difference in the results of SNG with deterministic number results in the conversion error, which in case of LFSR is at most 1/2^*k*^, since the all zero patterns is not generated in LFSR. The conversion is exact if the all zero state is artificially added in LFSR.

The correlation among SNs results in correlation-induced errors. In [18] a correlation factor, called SC correlation (SCC), is introduced to quantify the effect of correlation on the accuracy of SC. SCC between two stochastic numbers *A* and *B* is given in equation.

$$\begin{array}{c} SCC\left(A,B\right)=\{\begin{array}{ccc} \frac{P(A\wedge B)-P(A)P(B)}{\min(P(A),P(B))-P(A)P(B)} & P(A\wedge B)-P(A)P(B)>0, & \\ 0 & P(A\wedge B)-P(A)P(B)=0,\\ \frac{P(A\wedge B)-P(A)P(B)}{P(A),P(B)-\max(P(A)P(B)-1,0)} & otherwise \end{array} \end{array}$$ (1)

The SCC depends on the difference between the dot product and the real product of *A* and *B*. The difference is normalized by the maximum possible value to restrict SCC in the range of -1 to +1. The SCC of +1 and -1 indicates maximally correlated numbers, whereas, SCC = 0 means uncorrelated SNs. The authors in [18] have also given the function implemented by any two-input stochastic logic circuit based on SCC as mentioned in following equation.

$$\begin{array}{c} \delta^{{}^{\prime }}\left(A,B,SCC\right)=\{\begin{array}{cc} \left(1+SCC\right).\delta_{0}\left(A,B\right)-SCC.\delta_{-1}\left(A,B\right) & SCC<0,\\ \left(1-SCC\right).\delta_{0}\left(A,B\right)+SCC.\delta_{+1}\left(A,B\right) & otherwise \end{array} \end{array}$$ (2)

Where, *δ*_0_(*A*, *B*), *δ*_−1_(*A*, *B*), *δ*_+1_(*A*, *B*) are the functions implemented by two-input stochastic logic circuit in case of 0, -1 and +1 correlation, respectively, between SNs *A* and *B*. E.g. for two input AND gate, *δ*_0_(*A*, *B*) = *P*(*A*).*P*(*B*), *δ*_−1_(*A*, *B*) = max(*P*(*A*)+ *P*(*B*) − 1, 0) and *δ*_+1_(*A*, *B*) = min(*P*(*A*), *P*(*B*)). The above equation shows that the error increases linearly with increase in SCC. However, there are some exceptions, where the correlation has no effect on the functionality of the circuit. Two such exceptions for the case of SC-based FIR filters are discussed in section 3.

In this paper authors have focused on the correlation-induced errors in SC-based digital FIR filters.

## 3 Stochastic computing based digital finite impulse response filter

An *l*^*th*^ order FIR filter is represented as the inner product of the (*l* + 1)^*th*^-order input and coefficient vector:

$$\begin{array}{c} y_{n}=b_{0}x_{n}+b_{1}x_{\left(n-1\right)}+...+b_{l}x_{\left(n-l\right)}=\sum_{i=0}^{l}b_{i}x_{\left(n-i\right)} \end{array}$$ (3)

**Fig 3:**
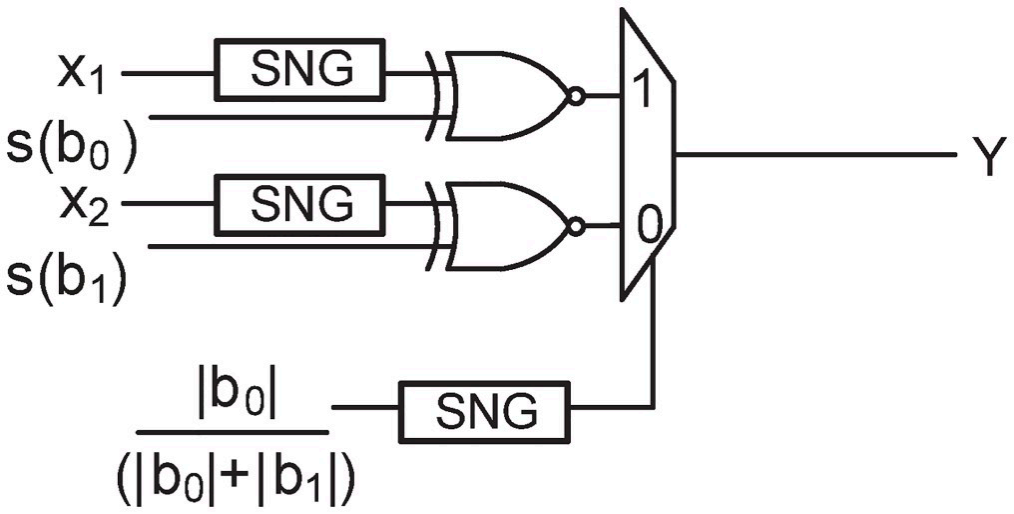
Stochastic inner-product circuit.

Where, *b*_0_…*b_l_* are the coefficients and *x_n_*…*x*_(*n*−*l*)_ are the current and *l* previous input samples given to the filter. As mentioned in previous section, these multiplication and addition operations can be implemented in SC with the help of AND (or XOR) gates and multiplexers (scaled-addition), respectively. In order to alleviate the effect of scaling in multiplexers, the authors in [5] have proposed an inner-product circuit, as shown in Fig 3. The inner-product circuit performs the following operation:

$$\begin{array}{c} s\left(b_{0}\right).\frac{|b_{0}|}{|b_{0}\left|+\right|b_{1}|}.x_{1}+s\left(b_{1}\right).\frac{|b_{1}|}{|b_{0}\left|+\right|b_{1}|}.x_{2}=\frac{1}{|b_{0}\left|+\right|b_{1}|}.\left(b_{0}.x_{1}+b_{1}.x_{2}\right) \end{array}$$ (4)

Where, *s*(*b*_*i*_) represents the sign of *b*_*i*_, which is 0 when *b*_*i*_ is positive and 1 when *b*_*i*_ is negative. The inputs *x*_1_ and *x*_2_ are real numbers in the range of -1 to +1. The inner-product circuit of [5] scales the addition according to the magnitude of the constant coefficients and therefore has better results than the conventional scaled addition circuit in SC. The above equation can be generalized for any *l*^*th*^ order FIR filter as follows:

$$\begin{array}{c} \frac{1}{|b_{0}\left|+\right|b_{1}\left|+...+\right|b_{l}|}.\left(b_{0}.x_{n}+b_{1}.x_{n-1}+...+b_{l}.x_{n-l}\right)=\frac{1}{\sum_{i=0}^{l}|b_{i}|}\sum_{i=0}^{l}b_{i}x_{\left(n-i\right)} \end{array}$$ (5)

and therefore, can be implemented from the combination of inner-product circuits containing *l*+1 XOR gates and *l* MUXes.

**Fig 4:**
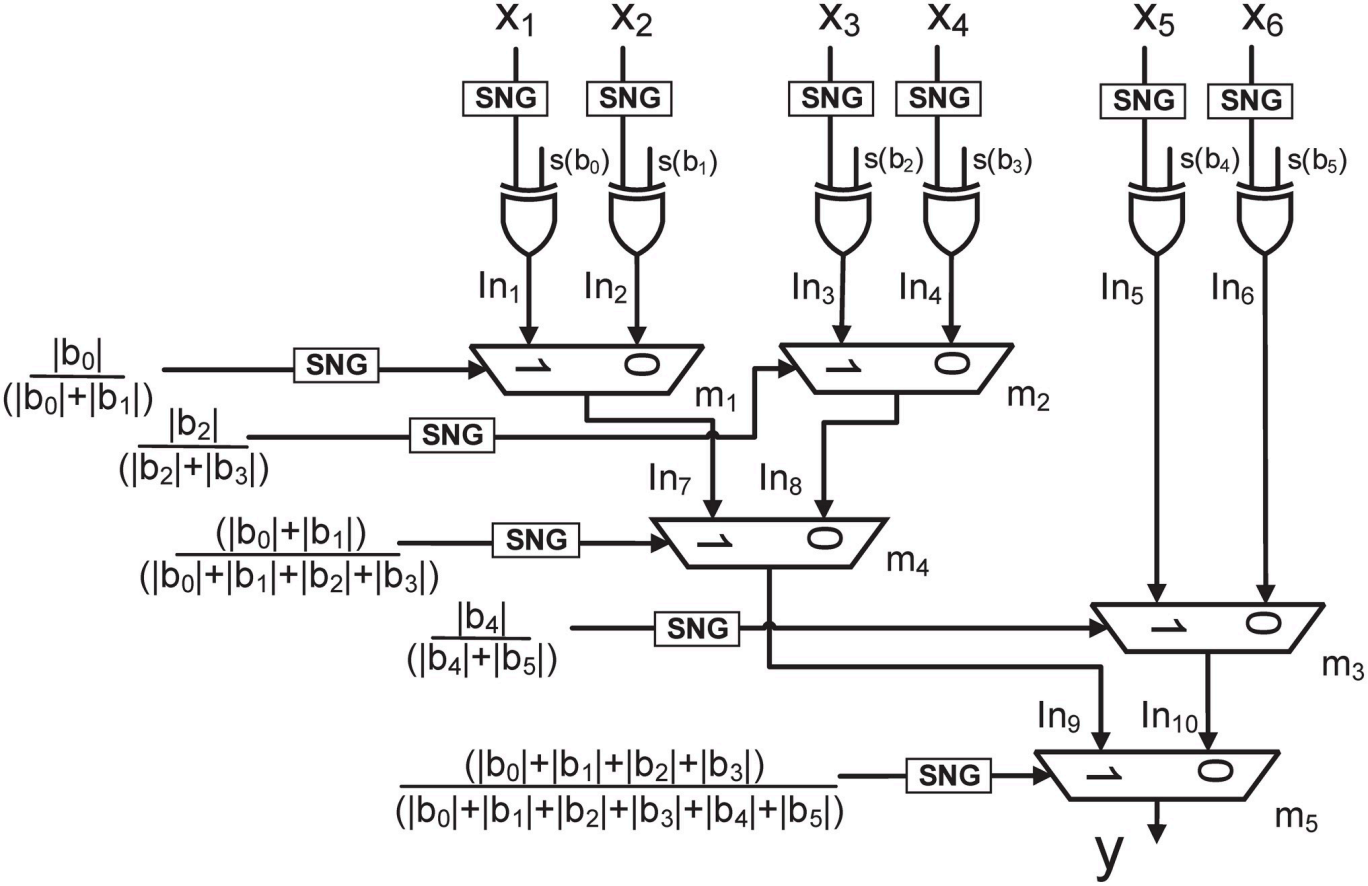
5^*th*^ order SC-based FIR filter.

Fig 4 shows an example of 5^*th*^ order FIR filter. The filter consists of 6 XOR gates for sign multiplications, a MUX tree consisting of 5 MUXes for implementing the scaled inner-product operation and 11 SNGs with distinct LFSRs for producing highly uncorrelated SNs for each input and select line of all MUXes. These high numbers of LFSRs, clearly, has a huge impact on the overall area of SC-based filters. However, as proposed in [8], an LFSR can be shared with different SNGs without affecting the overall accuracy of SC-based filters. The sharing of LFSR is based on the following two theorems [8]:

*Theorem-1*: The SCC between the two SNs applied at the data inputs of a MUX has no effect on the accuracy of the MUX output.
*Theorem-2*: The SCC between two SNs applied at the select lines of two different MUXes that are mutually unreachable (the output of one MUX does not reach the input of the other MUX and vice versa) does not affect the overall accuracy of the output of a MUX tree.

**Fig 5:**
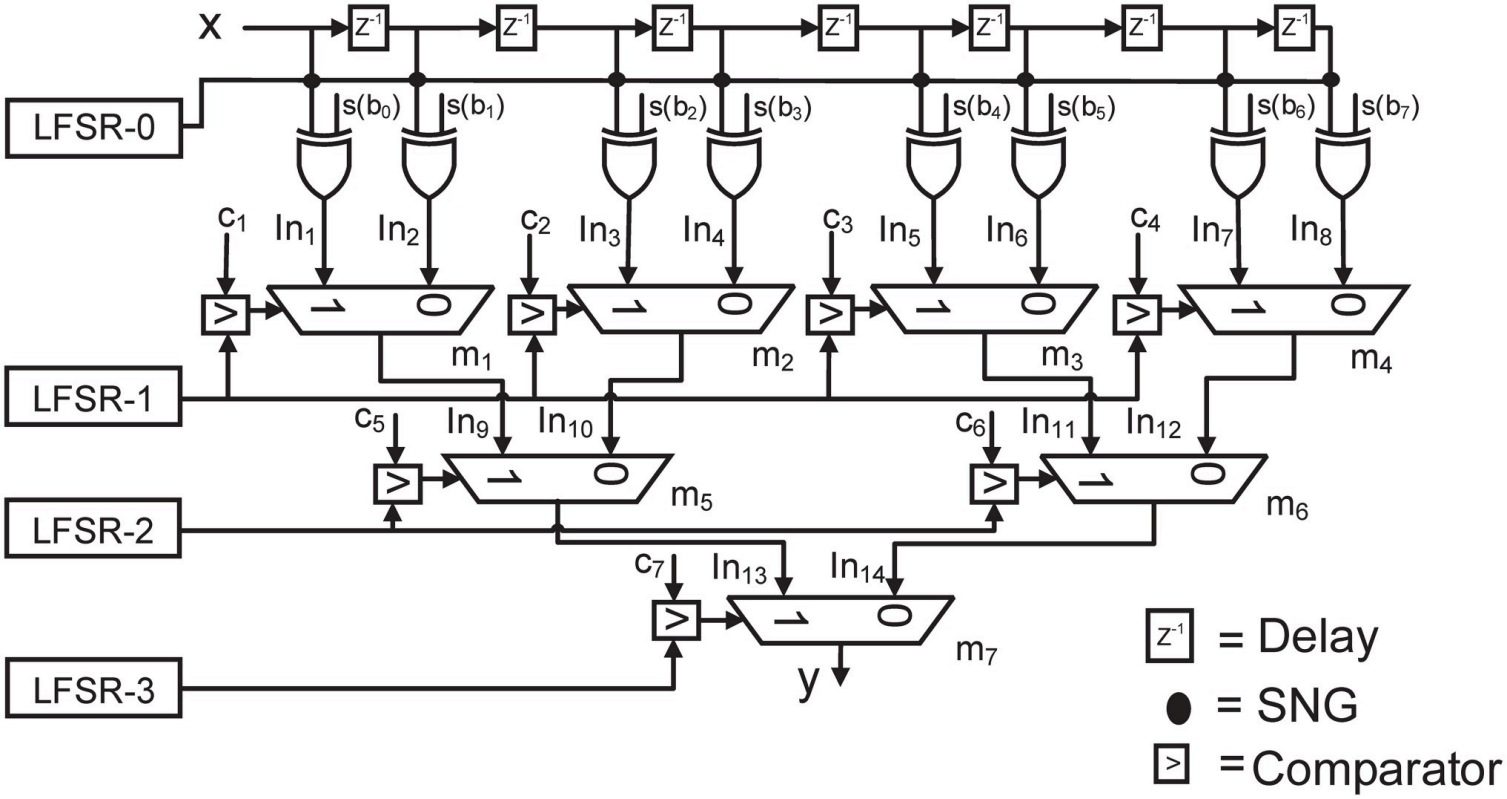
*Same-depth-share* architecture of 7^*th*^ order FIR filter [16].

The detailed proof derivations of these theorems can be seen in [16]. According to theorem-1, an LFSR can be shared with SNGs used for the stochastic conversion of all inputs of FIR filters. Similarly, according to theorem-2, an LFSR can be shared with SNGs used for the stochastic conversion of the values applied at the select line of MUXes that are mutually unreachable. This sharing greatly reduces the number of required LFSRs in SC-based filters. An example of the architecture of 7^*th*^ order FIR filter with shared LFSRs is shown in Fig 5. The architecture in Fig 5 is referred to as *same-depth-share* in [16], where, a tree like structure of MUXes is used with different stages and a single LFSR is shared among all MUXes of one stage. Similarly, a single LFSR is used for stochastic number generation of the current and the previous input samples. Therefore, the number of LFSRs required in this case is 4 as compared to 15, when each SNG has a distinct LFSR However, it should be noted that the number of LFSRs in *same-depth-share* increases with the increase in the order of FIR filters. In [16], a technique based on circular shift is proposed where only a single LFSR is used with its circularly shifted outputs with different shifts applied at all the SNGs used in the filter. This technique greatly reduces the area of SC-based filters, however, with some loss in accuracy as compared to *same-depth-share* case. Similarly, in [17] a permutation based strategy is proposed, where, the best permutations of a single LFSR are determined with reduced average SCC and applied at the select line of different MUXes. However, their methodology still achieves less accuracy as compared to the *same-depth-share* case. In fact as shown in results of [17] only a slight improvement as compared to *circular-shift* case is achieved by the permutation methodology. Moreover, the computational complexity for determining the best permutations is also large.

In this paper authors have explored the application of constant uniform patterns at the select lines of all MUXes with the aim of simplifying the area of SC-based filters and to maintain the same or higher accuracy as compared to the *same-depth-share* case.

## 4 Proposed methodology

In this section, the use of constant uniform patterns for the select line of MUXes in stochastic based FIR filters is discussed. The error performance is analyzed for 7^*th*^ order FIR filter by using different combinations of uniform patterns. The digital circuits are also designed for generating these uniform patterns.

### 4.1 Error performance analysis

As the filter coefficients and hence the values of select lines of MUXes in SC-based FIR filters are fixed once the filter is designed, therefore, the patterns of stochastic numbers for these select lines can be chosen in such a way as to reduce the complexity of hardware and to maintain the accuracy of filters by reducing the correlation between the values applied at the select lines. Therefore, in this work the possibility of applying uniform patterns are explored for these select lines, e.g. ‘11111....0000....00’ or ‘010101....00000....00’ or ‘00110011....00000....00’ etc. (pattern with 1’s in the beginning and 0’s in the end) and the shifted version of these e.g. ‘00000...11111....11’ etc., which are less complex to generate. It should be noted here that the patterns other than continuous 1’s and 0’s can only be applied in case where the value of select line is less than or equal to 0.5. The effect of using the mixture of these patterns on the error performance of different order FIR filters is analyzed. According to the theorems given in the previous section, the correlation error at the output of SC-based FIR filters depends upon two factors, 1) correlation between the select lines and the inputs of MUX and, 2) correlation between the select lines of different MUXes that are mutually reachable. The effect of these correlations for the case of uniform patterns is analyzed. The conventional and SC-based 7^*th*^ order FIR filter are implemented for error performance analysis. A floating point implementation of the conventional filter is done, whereas, for SC-based filters the input samples are represented as 8-bit fixed point numbers. Two cases of SC-based FIR filters are implemented, 1) *same-depth-share* case [8] and, 2) Uniform patterns case. For Uniform patterns case, the constant patterns are applied for all the select lines of MUXes and random patterns, using a single LFSR, for the inputs of filter. A set of 2000 samples of bird sound signal is applied to the filter.

Specifically, the different combinations of Uniform patterns that are analyzed are as follows:

*Comb-1*: A pattern of continuous one’s and zero’s i.e. ‘1111….0000….’ are applied to the select line of all MUXes and therefore, the correlation between the select lines is 1. Table 1 shows the average correlation between the inputs and select line of each MUX and the average absolute error at the output of each MUX. The error is measured by calculating the absolute difference at the output of each MUX from the floating point conventional filter and taking the average over all 2000 samples. For comparison, the results are also given for the *same-depth-share* case. The initial seed values of all LFSRs are different for the *same-depth-share* case. From the results it can be seen that the difference in average error for the first four MUXes in the first stage and the second stage is not very large although the average correlation between the select lines and the inputs is high for the uniform pattern case as compared to the *same-depth-share*. E.g. for m3, the average correlation for the *same-depth-share* case is 4.4E-5 and -4.1E-5, whereas, the average correlation for the uniform patterns case is -0.105 and 0.105, however, the difference in the average output error is only 7.3E-4 for the *same-depth-share* and uniform patterns case at m3. The average error at the last stage of MUX increases a lot for the uniform patterns case as compared to the *same-depth-share* case. It shows that the correlation between the select lines at higher stages has more adverse effect on the average error. The analysis was also done for different initial seed values of the LFSR(s) in *same-depth-share* and uniform patterns case and observed that the correlation between the inputs and select line of a MUX does not have a large effect on the average error at the output of the MUX. However, the average error at the last stage was more dependent on the correlation between the select lines at higher stages. Therefore, the patterns are selected in such a way as to minimize the correlation between the select lines at higher stages. In this regard two different combinations are applied which are given below.
*Comb-2*: *S*_1_, *S*_4_ and *S*_7_ = ‘00110011…..0000…..’, *S*_2_ and *S*_6_ = ‘111…..000….’, *S*_3_ and *S*_5_ = ‘010101….0000…..’.
*Comb-3*: *S*_1_ and *S*_3_ = ‘111…..000….’, *S*_2_ and *S*_4_ = ‘0000….1111….’, *S*_5_ = ‘010101….0000…..’, *S*_6_ = ‘0000…..010101…..’ and *S*_7_ = ‘0000….00110011…..’.

**Table 1:** Average correlation between the inputs and select line of each MUX and the average absolute error at the output of each MUX.

	*same-depth-share*		*Comb-1*		*Comb-2*		*Comb-3*	
	Avg-SCC (x10-3)	Avg-error (x10-3)	Avg-SCC (x10-3)	Avg-error (x10-3)	Avg-SCC (x10-3)	Avg-error (x10-3)	Avg-SCC (x10-3)	Avg-error (x10-3)
m1 (*In*_1_, *S*_1_)	-9.905	2.568	-59.5	3.787	216.1	2.99	-59.5	3.79
m1 (*In*_2_, *S*_1_)	9.904		59.56		-216		59.6	
m2 (*In*_3_, *S*_2_)	14.96	2.689	-152	3.948	-152	3.95	118	3.47
m2 (*In*_4_, *S*_2_)	-14.95		151.6		151.6		-118	
m3 (*In*_5_, *S*_3_)	0.044	2.753	-106	3.481	-57.2	3.88	-106	3.48
m3 (*In*_6_, *S*_3_)	-0.041		105.4		57.16		105	
m4 (*In*_7_, *S*_4_)	-7.969	2.509	-335	3.112	-160	3.58	39.8	3.66
m4 (*In*_8_, *S*_4_)	7.992		334.6		160.1		-39.8	
m5 (*In*_9_, *S*_5_)	-119.9	4.187	58.51	5.71	276.7	5.5	277	4.46
m5 (*In*_10_, *S*_5_)	119.7		-58.4		-277		-277	
m6 (*In*_11_, *S*_6_)	81.96	4.039	-272	5.174	-273	4.6	-5.01	4.79
m6 (*In*_12_, *S*_6_)	-81.64		270.9		272.4		4.09	
m7 (*In*_13_, *S*_7_)	-0.123	4.741	-71.1	12.35	92.48	8.9	-92.1	5.67
m7 (*In*_14_, *S*_7_)	-0.285		72.31		-91.8		92.2	

The correlation values between the select lines for these two combinations are given in Table 2. Table 1 provides the average error and average correlation results of each MUX for these two combinations. Table 1 shows that the average error at the output of last MUX is reduced in both of these combinations as compared to comb-1 due to less correlation between the select lines of MUXes. It should also be noted that although the correlation between the select lines of 1^*st*^ and 2^*nd*^ stage in comb-3 is high as compared to comb-2, however, the average error at the output of filter is lower for comb-3. This is due to the low values of correlation between the select lines of 2^*nd*^ and 3^*rd*^ stage of comb-3 as compared to comb-2. The analysis on comb-2 and comb-3 was also performed for different initial seed values of LFSR and it was observed that the average error at the last stage remained low for a wide range of different patterns of the filter input values.

**Table 2:** Correlation between selection lines of mutually reachable MUXes.

	Correlation between MUX select lines					
	SCC(*S*_1_,*S*_5_)	SCC(*S*_2_,*S*_5_)	SCC(*S*_3_,*S*_6_)	SCC(*S*_4_,*S*_6_)	SCC(*S*_5_,*S*_7_)	SCC(*S*_6_,*S*_7_)
**Same-Depth-Share**	0.024	0.074	-0.012	0.012	-0.03	0
***Comb-2***	0.39	-1	0.205	0.447	-0.03	0.067
***Comb-3***	-1	1	-1	0.605	0.034	0

### 4.2 Proposed architecture for uniform pattern generation

**Fig 6:**
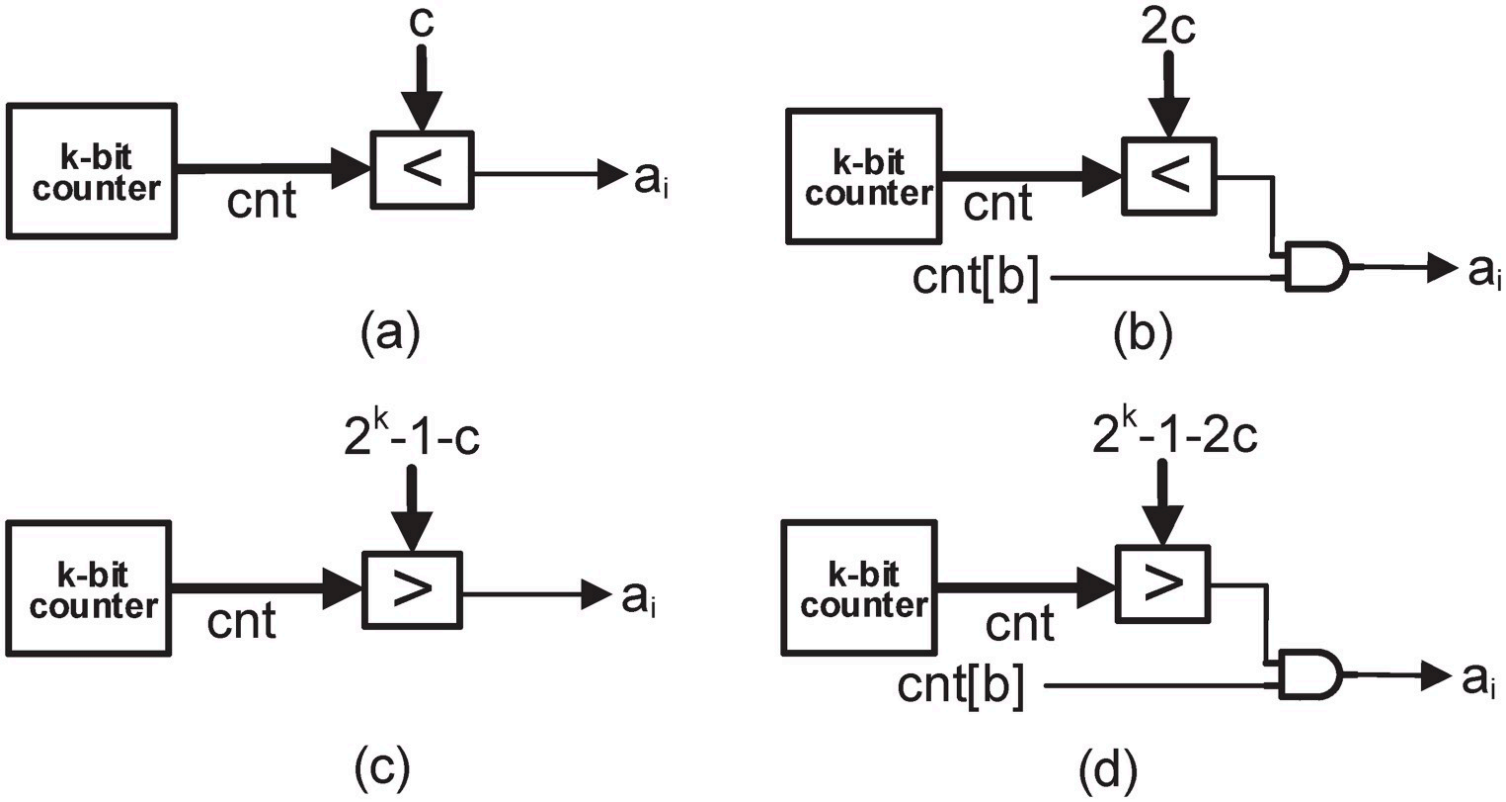
Proposed circuits for uniform pattern generation. (a) Circuit for generating a pattern of ‘1111…0000…’, (b) Circuit for generating rest of the patterns using a particular bit of counter, (c) and (d) Circuits for generating the Shifted version of the patterns in (a) and (b).

**Fig 7:**
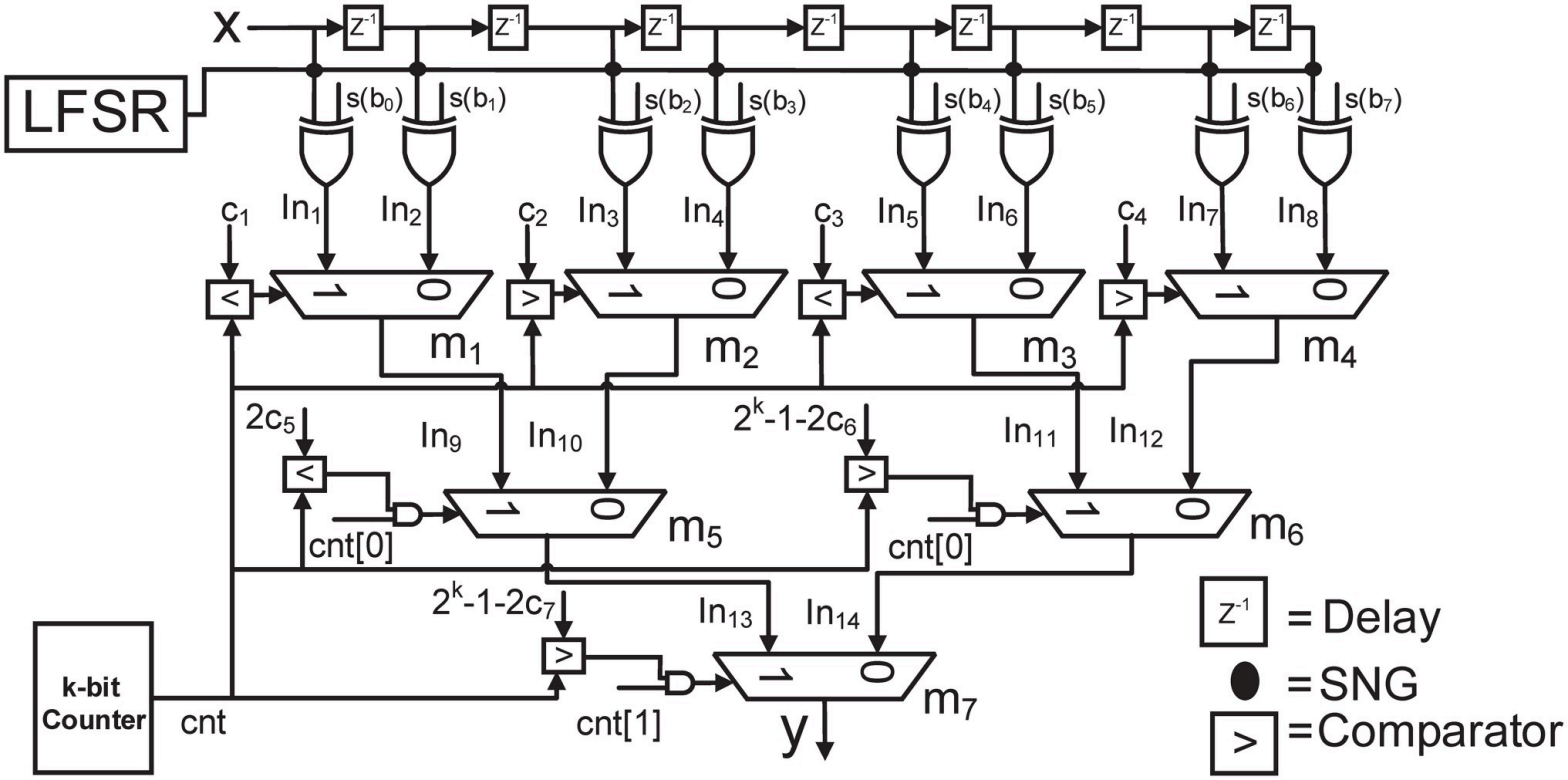
Proposed architecture for 7^*th*^ order FIR filter.

The proposed architecture for generating different uniform patterns as discussed in section 4.1 is shown in Fig 6. A single *k*−bit counter is used for generating 2^k^ bit stochastic numbers for select line of all MUXes. Fig 6(a) shows the architecture for generating the pattern of continuous ones and zeros i.e. ‘1111…0000…’, where, the comparator (the symbol ‘<’ means less than and the symbol ‘>’ means greater than) is used for comparing the value of the counter with a constant number c that has to be converted into the stochastic number *A* (where *a*_*i*_ is the *i*^*th*^ bit of *A*). As long as the value of counter is less than *c*, *a* 1 is produced at the output of comparator in each clock cycle and when it is greater than *c*, *a* 0 is produced at the output. Fig 6(b) shows the architecture for generating the rest of the patterns by using a particular bit of the counter (*cnt[b]*, 0≤b≤k-1) and an AND gate. E.g. *cnt[0]* will generate a pattern of ‘010101…0000…’, *cnt[1]* will generate a pattern of ‘00110011…0000…’ and so on. The AND gate will give the value of *cnt[b]* at the output as long as the output of comparator is true. When the output of comparator is false, the AND gate will give 0 at the output. The value applied to the comparator in this case is 2*c* instead of *c*, since for a particular value of *c*, the number of 1’*s* is completed in a particular bit of counter when the counter value is equal to 2*c*-1. The architectures for the shifted version of these patterns are shown in Fig 6(c) and 6(d), where 0’s are in the beginning and pattern with 1’s are shifted at the end (pattern with 1’s start when the counter value is greater than 2*^k^* −1−*c* or 2*^k^*−1−2*c*). The overall architecture of 7^*th*^ order FIR filter for the case of *comb-3*, as given in section 4.1, is shown in Fig 7. From the figure can see that proposed architecture only requires 1 LFSR, a single *k*−bit counter and some additional AND gates as compared to 4 LFSRs for the *same-depth-share* case. It should be noted that the number of LFSRs increases for high order filters in *same-depth-share* case, e.g. 6 LFSRs are required for 15^*th*^ order FIR filter. However, in proposed architecture only a single LFSR and a single counter is required, irrespective of the filter order, and therefore, the area is reduced further for high order filters as compared to the *same-depth-share*.

## 5 Results

**Fig 8:**
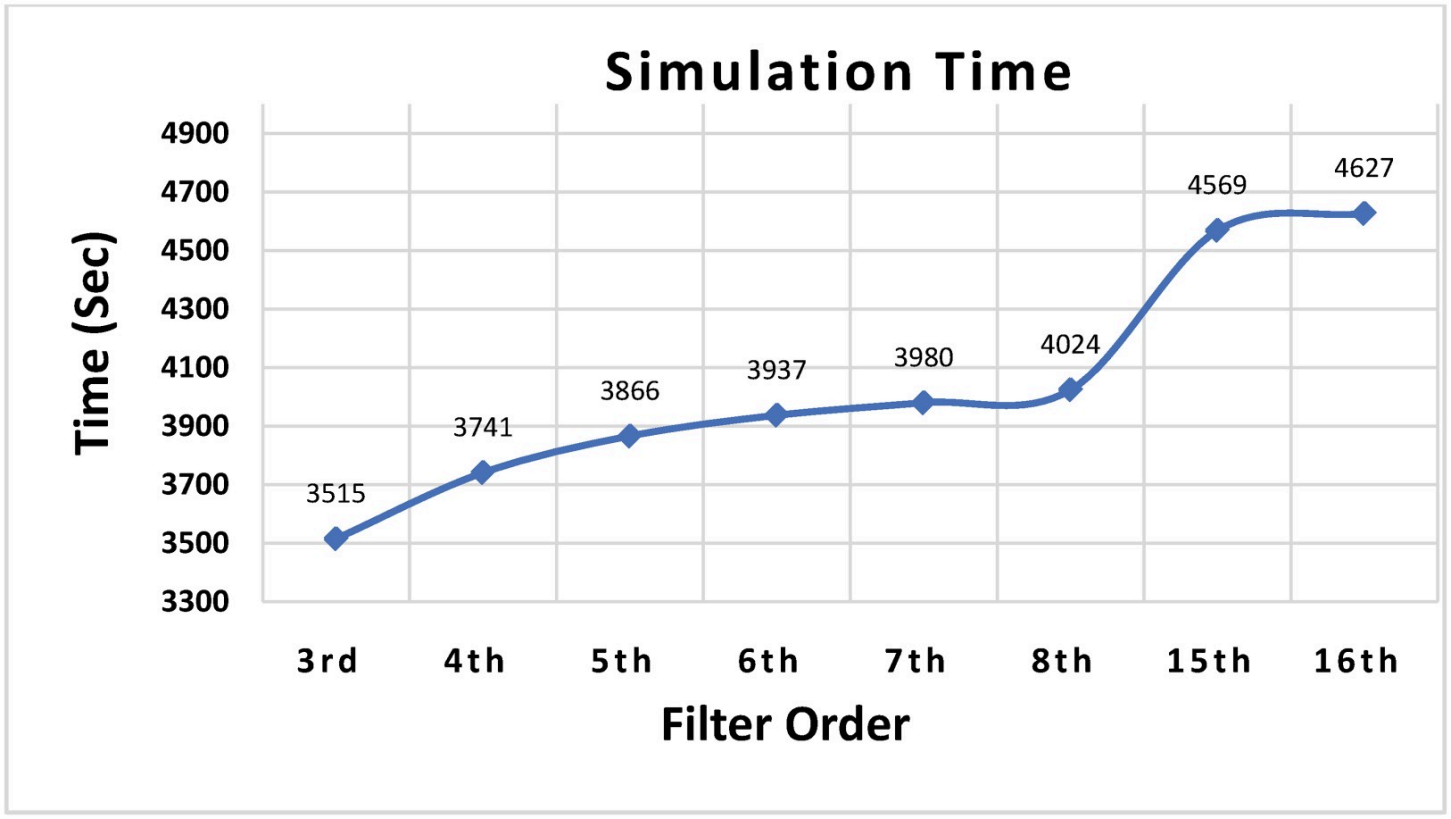
Simulation time of proposed algorithm for different order SC-FIR filters (50 different seed values of LFSR with 2000 input samples for each seed value, a total of 100,000 attempts for each order).

To evaluate the effectiveness of the proposed uniform patterns-based approach, FIR filters of several orders are implemented in MATLAB. The simulations are performed on a Intel Core (TM) i7-8700 @ 3.20 GHz processor with 32 GB RAM. The best uniform patterns for different filters in the proposed methodology are selected based on the strategy discussed in section 4.1. For comparison, the same filters are also implemented for the *same-depth-share* case and *all-cs-3* case (*all-share* case) proposed in [16], and the permutation based methodology proposed in [17], herein this paper referred to as *all-perm-share*, which are best available implementations in the literature in terms of error performance and hardware resources. In *all-cs-3* case, a single LFSR is used with its circularly shifted outputs applied at different stages of MUXes, where, the difference in shift amount is 3 between each consecutive stage of MUXes. The input samples and the values of select lines of MUXes are represented as 8-bit fixed point numbers and hence an 8-bit LFSR and counter is used for converting the 8-bit input samples and select lines of MUXes into SNs. We took 50 sets of initial seed values of LFSR(s) for all cases and applied 2000 input samples to a filter for each set. The seed values in each set are chosen randomly. Therefore, a total of 100,000 attempts are made for each filter and the average absolute output error is calculated from these attempts. The total simulation time of 100,000 attempts for all implemented FIR filters in this work is shown in Fig 8. From the figure can see that the simulation time grows logarithmically with the filter order. This is due to the logarithmic increase in the number of stages of MUXes with the increase in filter order. Whereas, the computational complexity grows linearly with the increase in the number of input samples for each order FIR filter. Table 3 shows the results of average absolute output error for all implemented filters. From the table can see that proposed design has less average error as compared to *same-depth-share* for all order FIR filters, and especially, for high order FIR filters. E.g. For 15^*th*^ and 16^*th*^ order FIR filters the average error is 17.7% and 19.6%, respectively, less than the *same-depth-share* case. The less error is achieved due to the careful analysis of the impact of different uniform patterns (with different SCC values) on average error at different stages of MUXes and choosing the best possible patterns for each filter order with reduced SCC values, especially, at the later stages of MUXes. Similarly, proposed design strategy outperforms *all-cs-3* and *all-perm-share* methodology for all order FIR filters with up to 68.2% and 45.8% reduction in average error, respectively. This is due to the fact that different uniform patterns are chosen at different stages of MUXes as compared to the circular shifts or permutations of a single LFSR in previous approaches. The simulation results show the effectiveness of the proposed design methodology in terms of error performance.

**Table 3:** Comparison of average absolute output error (x10^−3^) for different order FIR filters.

Filter Order	*Same-Depth-Share* [16]	*all-cs-3* [16]	[17]	*This Work*
3rd	4.63 (33.8%)	13.71	9.26(67.54%)	**4.36 (31.8%)**
4th	5.97 (52.8%)	11.31	10.49(92.75%)	**5.30 (46.9%)**
5th	6.30 (60.6%)	10.39	8.08(77.77%)	**6.06 (58.3%)**
6th	6.14 (63.6%)	9.65	8.47(87.77%)	**6.01 (62.3%)**
7th	7.11 (52.5%)	13.55	10.23(75.49%)	**7.05 (52.0%)**
8th	54.5 (96.7%)	56.34	55.70(98.86%)	**52.7 (93.5%)**
15th	10.1 (87.4%)	11.56	10.95(94.72%)	**8.31 (71.9%)**
16th	11.1 (66.9%)	16.6	10.93(65.84%)	**8.93 (53.8%)**

For evaluating the area of the proposed design, implemented 3^*rd*^, 5^*th*^, 7^*th*^ and 15^*th*^ order FIR Filters in Verilog HDL for the *same-depth-share*, *all-cs-3*, *all-perm-share* and uniform patterns case. Xilinx Virtex-7 XC7VX485T FPGA device is used for implementing these filters. Table 4 shows the hardware resources for all implemented filters where, the hardware resources for *all-cs-3* and *all-perm-share* case are same and presented in a single column. From the table we can see that the hardware resources of the proposed work are comparable to the *all-cs-3* and *all-perm-share* case. There is a difference of 8 flip-flops between proposed work and *all-cs-3*/*all-perm-share* case for all order FIR filters due to the use of an additional 8-bit counter for generating the uniform patterns for all the select lines of MUXes. The hardware resources are less as compared to *same-depth-share* case, especially, for higher order filters. E.g. for 15^*th*^ order FIR filter, reduction in 6-input Lookup Tables (LUTs) and flip-flops (FFs) is 19% and 15%, respectively.

**Table 4:** Resource utilization on Xilinx Virtex-7 XC7VX485T FPGA.

Filter Order	*Same-Depth-Share* [16]				*all-cs-3* [16] & [17]				*This work*			
	6-input LUT	FF	F7 MUX	F8 MUX	6-input LUT	FF	F7 MUX	F8 MUX	6-input LUT	FF	F7 MUX	F8 MUX
3^*rd*^	24	48	0	0	25	32	1	0	28	40	0	0
5^*th*^	37	72	0	0	34	48	0	0	33	56	1	0
7^*th*^	49	88	1	0	43	64	2	1	44	72	0	0
15^*th*^	90	160	3	1	68	128	3	0	73	136	0	0

## 6 Conclusion and future work

In this paper, the application of constant uniform patterns on the select lines of MUXes is investigated for SC-based digital FIR filters. The error performance of these patterns is analyzed and the combinations of these patterns are proposed for optimizing the error performance. The architectures for generating these constant uniform patterns are also proposed. The proposed architecture requires only 1 LFSR and 1 counter for generating the SNs for all the inputs and select lines of filter, irrespective of the filter order. Results show that the proposed strategy has better error performance and comparable hardware complexity as compared to the best available implementations in the literature.

The proposed methodology uses an additional counter for generating the uniform patterns which results in some increase in area as compared to the state-of-the-art methods. Future work is directed towards further reducing the area/power consumption of the proposed method and reducing the latency of the SC-based filters, while maintaining the same accuracy.
